# Eye movements during the Iowa Gambling Task in Parkinson’s disease: a brief report

**DOI:** 10.3389/fpsyg.2025.1478500

**Published:** 2025-04-04

**Authors:** Kirby Doshier, Anthony J. Ryals, Vicki A. Nejtek, Michael F. Salvatore, Jerome Lisk

**Affiliations:** ^1^Department of Psychology, University of North Texas, Denton, TX, United States; ^2^University of North Texas Health Science Center, Fort Worth, TX, United States; ^3^Movement Disorders, Denton, TX, United States

**Keywords:** Parkinson’s disease, eye tracking, Iowa Gambling, decision-making, eye blinks

## Abstract

Parkinson’s disease (PD) is characterized by motor and cognitive impairments. Subtle cognitive impairment may precede motor impairment. There is a substantial need for innovative assessments, such as those involving decision-making, to detect PD in the premotor phase. Evidence suggests executive dysfunction in PD can impede strategic decision-making relying on learning and applying feedback. The Iowa Gambling Task (IGT), when combined with eye-tracking, may be a valuable synergistic strategy for predicting impaired decision-making and therapeutic non-compliance. Participants with PD and matched healthy controls completed the Movement Disorders Society’s modified Unified Parkinson’s Disease Rating Scale (UPDRS-MDS), 6-min Walk Test (6MWT), Timed Up and Go Test (TUG), Trail Making Test A and B (TMT A and B), Controlled Oral Word Association Test (COWAT), and the Barratt Impulsiveness Scale (BIS). Eye tracking was recorded during the IGT. The PD group scored significantly higher on UPDRS subscales and travelled less distance during the 6MWT despite equivalent performance on the TUG. The PD group also had longer completion times on TMT A and B and more errors on TMT B. Overall IGT winning scores were marginally worse in PD. However, when analyzed as a function of performance over time, the PD group performed significantly worse by task end, thus suggesting impaired decision-making. PD participants exhibited a 72% reduction in blinks despite equivalent outcomes in other eye-movements. Combined with established motor and executive function tests, the inclusion of eye-tracking with the IGT may represent a powerful combination of noninvasive methods to detect and monitor PD early in progression.

## Introduction

Parkinson’s disease (PD) is characterized by distinct motor dysfunctions; bradykinesia, tremors, and rigidity, that are associated with degeneration of dopaminergic neurons in the substantia nigra leading to a loss of dopamine signaling (Kordower et al., 2013; Kouli et al., 2018; Wirdefeldt et al., 2011). Importantly, non-motor symptoms occur in roughly 90% of PD patients (McDowell and Chesselet, 2012) with a prodromal period including depression, anxiety, and cognitive difficulties preceding motor symptoms (Durcan et al., 2019). Notably, up to 50% of PD patients experience a form of mild cognitive impairment characterized by subtle cognitive changes not always apparent on standardized tests (Goldman and Litvan, 2011). These changes may directly affect quality of life including through therapeutic non-compliance (Straka et al., 2019; Daley et al., 2012). Some have proposed that cognitive deficits can be present up to 10 years before PD diagnosis (Fengler et al., 2017). Because the subtleties in cognitive dysfunction in PD are under-detected and under-treated in clinical practice, it is highly important for skilled clinicians to recognize changes in cognitive domains that are risk factors for early decline. To aid in such recognition, a need exists for techniques combining decision-making behavior with psychophysiological correlates, such as eye tracking, which may enhance the characterization and early diagnosis of PD (Antoniades and Spering, 2014).

One cognitive domain particularly vulnerable to early PD-related decline is executive functioning. Executive functioning is essential for goal-directed behavior and adjustments to novel situations (Nejtek et al., 2021; Friedman and Miyake, 2017). The prefrontal cortex (PFC) is critically involved in executive functioning, such as through selection and inhibition (Aron et al., 2004; Dulas and Duarte, 2014; Dulas and Duarte, 2016), conflict monitoring (Botvinick et al., 2004) and risk and reward processing (Bechara et al., 1994; Bechara and Damasio, 2005). In line with this, individuals with PD often exhibit impairments in evaluating reward-based outcomes (Perugini et al., 2018), they display a tendency toward risk taking, and they struggle to learn from negative feedback (Colautti et al., 2021).

The Iowa Gambling Task (IGT) is one of the most widely used assessments for understanding decision making abilities under conditions of reward and uncertainty in individuals with PD (Colautti et al., 2021; Salvatore et al., 2021). Salvatore et al. (2021) propose that the IGT may be sensitive to detecting prodromal changes due to its close relationship with several key regions involved in the neurological dysfunction of PD patients (e.g., frontal cortex and locus coeruleus). Many studies have reported significantly lower IGT performance in PD patients compared to controls (e.g., Salvatore et al., 2021; Castrioto et al., 2015; Xi et al., 2015), perhaps reflecting an inability to balance reward and risk (Kobayakawa et al., 2010; Kobayakawa et al., 2017). Neurobiologically, these deficits may stem from a substantial loss of dopaminergic function in the midbrain (e.g., substantia nigra and ventral tegmental area) and noradrenergic dysfunction in the locus coeruleus, both of which have extensive projections to the prefrontal cortex (Salvatore et al., 2021; Alberico et al., 2015; Mather and Harley, 2016). Accordingly, some studies have estimated a 40–77% loss of dopamine-based innervation in the ventral tegmentum and a 63% cell loss in norepinephrine-based cell loss in locus coeruleus in patients with PD (Alberico et al., 2015; Mather and Harley, 2016).

Given that eye movements are closely linked to both dopamine and norepinephrine signaling status, eye tracking offers a promising and noninvasive window into the neural mechanisms underlying cognitive decline in PD. Fixations, saccades, and blinks are robust reflections of both perceptual and cognitive functions (Spering, 2022), and multiple mid-brain and frontal connections are involved in eye movements (McGinty et al., 2016; Ding et al., 2009; Hui and Beier, 2022). For instance, dopaminergic activity involved in learning, memory, and goal-oriented behavior can be indirectly measured through spontaneous eye blink rate during visual exploration (Van Slooten et al., 2019). Specifically, dopamine is thought to modulate the frequency of these spontaneous eye blinks involved in reward-driven behavior and cognitive flexibility (Jongkees and Colzato, 2016). Eye blinks are also related to dopaminergic activity in basal ganglia by contributing to both motor and cognitive functioning (Willett et al., 2023; Redgrave et al., 2010; Eckstein et al., 2017; Zhou et al., 2021). Understanding how cognitive deficits manifest through eye movement abnormalities may provide valuable early indicators of neurodegeneration (Wong et al., 2018). Unfortunately, research linking decision-making deficits with eye movement abnormalities in early PD remains limited (Wong et al., 2018; Archibald et al., 2013; Wang et al., 2016; Tsitsi et al., 2021; Ranchet et al., 2017). To our knowledge, no study has integrated the IGT with eye tracking to examine decision-making in individuals with early-stage PD. This novel approach may provide deeper insight into the neurocognitive mechanisms underlying PD-related cognitive decline and could offer a valuable tool for early disease detection.

The first goal of the present study was to test motor functioning, verbal fluency, impulsivity, and cognitive flexibility in a sample of early-stage PD patients evaluated during their “ON” state compared to age-matched and education-matched healthy controls. Our second goal was to record eye movements during completion during decision-making using the IGT. We first hypothesized that participants with PD would perform worse on motor tasks and measures of attention and cognitive flexibility compared to healthy controls. Second, we hypothesized that the number of fixations, fixation durations, and number of eye blinks, would significantly differ between individuals with PD and healthy controls during the IGT. Finally, we hypothesized that overall behavioral performance on the IGT would be worse for our PD group.

## Method

We secured Institutional Review Board approval through the University of North Texas on February 26th, 2024, prior to beginning this study (Reference Number IRB-23-425). Participants were recruited through community-based and non-profit organizations, healthcare providers, local businesses, universities, and word of mouth. Prospective participants were given a brief overview of the motor tasks and a general overview of the cognitive assessments used. Examinations occurred in the Neurocognitive Laboratory at the University of North Texas and were directly observed by the primary investigator with the length of each assessment ranging from 45 min to 150 min depending on clinical status of the participant.

### Experimental power consideration

An *a priori* power analysis was conducted using IBM SPSS version 29.0.0.0. Results indicated the required sample size to achieve 80% power for detecting a medium effect, at a significance criterion of *α* = 0.05, was a total of *N* = 24 for repeated measures analysis of variance including within and between-subjects interactions.

### Inclusion criteria for PD subjects and matched healthy controls

Inclusion criteria included English-speaking/reading/writing individuals between 40–90 years old who self-identified as Black/African American, Non-Hispanic White, and Hispanic with a minimum high school grade level education or General Education Development (GED). Healthy controls had to perform within existing normative ranges on cognitive tests. A diagnosis of PD was verified during in-person screening with the Movement Disorder Society (MDS) Clinical Diagnostic Criteria from the Unified Parkinson’s Disease Rating Scale (UPDRS). These diagnoses were confirmed by a board-certified neurologist through a private practice as a movement disorder specialist. Each participant received a $25 gift card as compensation. Exclusion criteria included current autoimmune, metabolic, endocrine, neurological, or psychiatric disorders, self-reported illicit substance use, pregnancy, or substantial visual impairment. Individuals currently taking psychoactive medications were excluded, and participants were asked to abstain from alcohol and tobacco prior to the study.

### Participants and procedure

Our final sample consisted of 25 individuals with early-stage PD (Mean age: 73.0; 7 females) and 20 healthy age-matched and education-matched controls (Mean age: 69.6; 16 females). Individuals with PD presented at a mean Hoehn and Yahr (1967) Stage of 1.44 indicating unilateral and unliteral/axial movement involvement (see Table 1). Upon completion of informed consent, participants completed a basic demographic questionnaire. Blood pressure (systolic/diastolic) and heart rate were then taken, and the Ishihara Test for Color Blindness (Clark, 1924) was administered to screen for color-deficiencies (none were observed). Assessments were then given in the following order.

**Table 1:** Participant demographics.

	PD group (*N* = 25)	HC group (*N* = 20)
Characteristic	Mean (SD)	Mean (SD)
Age (years)	73.0 (6.3)	69.6 (6.3)
Gender (M/F)	18/7	4/16
Education (years)	15.2 (2.7)	16.6 (2.5)
Years since PD Dx	4.7 (4.4)	
Hoehn and Yahr Stage	1.44 (0.53)	

PD, Parkinson’s disease; HC, healthy control; Dx, diagnosis. Groups did not differ between years of education based upon independent samples *t*-tests: [*t* (43) = 1.82, *p* = 0.08] or age [*t* (43) = 1.64, *p* = 0.10].

### UPDRS

The UPDRS was administered to participants to determine stage of PD using the Movement Disorders Society recommended guidelines using four fundamental diagnostic criteria categories: I. Mentation, Behavior, and Mood, II. Activities of Daily Living, III. Motor Examination, and IV. Complications of Therapy (in the past week). Questions were asked relative to each section in parts I–III, while motor function was assessed visually and physically by the investigators in part IV. Parts I–III were scored using a rating scale of 0–4 per item, while part IV uses yes/no indications (Goetz et al., 2008).

### Motor tasks

The 6MWT measured travel distance using self-selected gait speed over 6 min via stopwatch. Start and stop positions were marked in an empty hallway 30 meters apart using tape. The TUG assessed the amount of time it takes for the participant to get up from a seated position and walk 10 feet at a self-selected gait speed. Start and stop positions were marked using tape.

### Trail Making-Task (TMT-A and B)

The TMT-A measured attention, visual search, processing speed, and basic motor coordination through sequence following. The TMT-B measured attention, visual search, sequence following, cognitive flexibility, and set-shifting ability (Reitan and Tarshes, 1959). Participants performed the TMT-A and B test versions using pencil and paper. The TMT-A required participants to draw individual lines sequentially connecting 25 encircled numbers randomly positioned on an 8 × 11 inch test sheet. The TMT-B required participants sequentially connect alternating letters and numbers (e.g., 1, A, 2, B, 3, C, etc.). These tasks were scored based on total completion time and number of errors.

### Barratt’s Impulsiveness Scale

The Barratt’s Impulsiveness Scale (BIS) was a self-report questionnaire used to assess the tendency to act without considering consequences by evaluating aspects like cognitive response speed, lack of impulse control, and risk-taking behavior. Participants were asked 30 Likert-type questions regarding impulsive tendencies with answers ranging from 1 (rarely/never) to 4 (almost always/always) (Patton et al., 1995). Items were scored based on six primary factors: (attention, motor, self-control, cognitive complexity, perseverance, and cognitive instability) and three second-order factors (attentional, motor, and non-planning). A total composite score was used in analyses with higher scores indicating higher levels of impulsivity.

### Controlled Oral Word Association Test

The Controlled Oral Word Association Test (COWAT) was used to measure verbal fluency, which is the ability to produce words spontaneously. It included two phases: Animal Naming and FAS (Benton et al., 1983). For Animal Naming, participants were given 60 s to name as many animals as they could think of as quickly as possible. For the FAS test, participants completed three separate 60 s trials where they were asked to orally produce words that begin with the letters F, A, and S as quickly as possible.

### Iowa Gambling Task

The Iowa Gambling Task (IGT) was used to measure decision-making skills under conditions of uncertainty and risk and to gauge how people learn to make profitable decisions over time. Participants completed a computerized version of the IGT. Stimuli were presented centrally using E-Prime 3.0 on a 66 cm LCD monitor set to a 1,204 × 768-pixel resolution and a 250 Hz refresh rate. The IGT consisted of 100 card selection trials from four identical decks of cards (A, B, C, and D). Participants chose cards one at a time by using a mouse to select their preferred deck. Each time they chose a card, they were given feedback on whether they received a gain or loss of funds based on their choice, and “money” was deposited or debited from their account accordingly. All participants started with an amount of $2,000 and were told to make a profit. They had no prior knowledge about the amount that the chosen card would yield. Decks A and B always yielded $150, while decks C and D always yielded $50. For each card chosen, there was a 50% chance of having to pay a penalty. For decks A and B, the penalty was $250, whereas for decks C and D it was $50. This task was self-paced and lasted approximately 10 min. The IGT critically depends on calculating a cumulative tally of wins vs. losses over time, and its continuous nature allows participants to learn and adapt their decision-making strategies over time based on feedback.

### Eye movements during the IGT

Eye movements (fixation, saccades, and blinks) were recorded continuously from the right eye using an Eye Link 1000-Plus tracker with a 250 Hz sampling rate and controlled ambient lighting. Participants were comfortably seated 30 cm (about 11.81 in) from the screen, and their heads were stabilized with a chinrest to minimize movement. Once comfortable, the camera lens was focused, and participants’ eyes were calibrated to the participant’s computer screen (mirrored to the experimenter’s computer screen) and validated using a multipoint grid. After calibration, participants were prompted to begin the IGT, and computerized instructions were given while a researcher remained in the testing room. Recording began with the presentation of the first stimulus and continued without interruption as participants made their choices, received brief feedback, and proceeded through each trial. Recording ceased only after all 100 trials were completed. For analysis, averages for each type of eye movement were taken for each trial block (20 trials) across all trial (100 total).

### Statistical analyses

We used IBM SPSS v.29 for all analyses, alpha was set at *p* < 0.05, and effect sizes are reported. Performance on UPDRS, motor assessments, TMT A and B, BIS, COWAT, IGT, and eye movement measures were analyzed using a series of one-way ANOVAs to test for between-groups differences. We also conducted a 2 between (PD, Control) × 5 within (block) mixed repeated measures ANOVA under the general linear model (GLM) framework in SPSS to assess learning and decision-making over time. For each outcome variable of interest, we conducted maximum normalized residual analyses to identify extreme values and report as outliers that could bias results (Grubbs, 1969). We also used exploratory partial least squares correlations between our eye fixation duration results and IGT blocks to shed light on learning behaviors.

## Results

### Unified Parkinson’s Disease Rating Scale

To assess the differences in Parkinson’s disease symptoms among the two groups (PD group and healthy control group), we used summarized subscale scores from the three sections of the Unified Parkinson’s Disease Rating Scale (UPDRS): Part I (Mentation, Behavior, and Mood), Part II (Activities of Daily Living), and Part III (Motor Examination). As expected, results revealed significant group effects for each of the three subscales with PD patients scoring higher for each outcome measure (listed in Table 2).

**Table 2:** UPDRS between-groups comparisons.

	PD Group	HC Group			
Characteristic	Mean (SD)	Mean (SD)	*F*	*p*	*η* ^2^
UPDRS Part I	9.00 (5.15)	2.70 (2.36)	25.82	**<0.001**	0.37
UPDRS Part II	9.48 (7.51)	0.50 (0.76)	28.22	**<0.001**	0.39
UPDRS Part III	21.5 (15.03)	1.20 (3.65)	40.21	**<0.001**	0.48
6-min Walk (m)	300.80 (97.6)	365.68 (67.2)	6.40	**0.01**	0.13
TUG (s)	8.18 (5.7)	7.19 (1.7)	0.57	0.45	0.01
Gait (m/s)	0.98 (0.4)	1.05 (0.3)	0.53	0.47	0.01

UPDRS Part I, Mentation Scores; Part II, Activity of Daily Living; Part III, Motor Examination; TUG, Timed Up-and-Go; m, meters; s, seconds; m/s, meters per second. Bold values represent statistically significance group differences based upon the threshold of *p* <0.05.

### Motor assessments

Distance and speed were analyzed for the three different motor function assessments (6-min Walk Test, TUG test, and Gait Speed) (listed in Table 3). Results revealed a significant group difference indicating that individuals with PD walked a shorter duration than healthy controls in 6 min. For the TUG analysis, four individuals were identified as outliers due to scoring difficulties with timing errors. Therefore, as these outliers were excluded from the analyses, this comparison was conducted on *N* = 41 (21 PD, 20 Matched Controls). TUG and Gait Speed analyses revealed no significant between-groups differences (see Table 2).

**Table 3:** Cognitive and IGT test results.

	PD group	HC group			
Test	Mean (SD)	Mean (SD)	*F*	*p*	*η* ^2^
TMT-A (RT)	38.09 (14.7)	27.48 (6.5)	9.08	**0.004**	0.18
TMT-A (errors)	0.44 (0.9)	0.20 (0.4)	1.29	0.26	0.03
TMT-B (RT)	100.62 (56.1)	58.81 (15.2)	10.45	**0.002**	0.20
TMT-B (errors)	1.72 (2.1)	0.35 (0.6)	7.76	**0.008**	0.15
BIS	55.96 (7.9)	54.70 (9.7)	0.23	0.63	0.01
FAS	37.92 (13.6)	41.70 (12.5)	0.92	0.34	0.02
Animal Naming	22.08 (5.3)	22.40 (5.4)	0.04	0.84	0.00
IGT	0.13 (15.28)	8.10 (14.49)	3.11	0.08	0.07
IGT-1 (1–20)	−0.08 (2.91)	−2.40 (6.31)	2.68	0.11	0.06
IGT-2 (21–40)	0.64 (7.02)	1.80 (3.94)	0.44	0.51	0.01
IGT-3 (41–60)	1.28 (5.71)	0.70 (3.85)	0.15	0.70	0.00
IGT-4 (61–80)	1.84 (6.66)	3.70 (7.90)	0.74	0.40	0.02
IGT-5 (81–100)	−0.32 (5.68)	4.50 (5.94)	7.69	**0.008**	0.15

TMT, Trail-Making Task; BIS, Barrett Impulsiveness Scale; FAS, COWAT Verbal Fluency Score; Animal Naming, COWAT Naming Score; IGT, Iowa Gambling Task. IGT 1–5 refers to the block number and trial numbers within that block. Bold values represent statistically significance group differences based upon the threshold of *p* <0.05.

### Cognitive assessments

#### Trail Making Test (TMT A & B)

Results for all cognitive assessments are listed in Table 3. Outlier analyses identified two extreme values for TMT A and TMT B in the PD group, thus results are reported for *N* = 23 and *N* = 20, respectively. Between-groups differences in mean completion times (seconds) were significant for both TMT A and TMT B, with healthy controls completing both tests faster than the PD group. The average number of errors did not differ between groups for version A. However, PD participants exhibited a significantly higher number of errors than controls in version B. A change analysis (mean time to complete Trail B minus time to complete Trail A) between groups supported poorer performance in PD participants [*t* (41) = 3.04, *p* = 0.004, *d* = 0.90]. Taken together, these results indicates that those with early-stage PD need a longer amount of time to process information that requires cognitive flexibility and shifting attention—even with familiar letters and numbers.

#### Barratt Impulsiveness Scale

A between-groups analysis of the BIS revealed no significant differences between individuals with PD and healthy controls. Additionally, a series of one-way ANOVAs examining the three BIS subdimensions—attention impulsivity, motor impulsivity, and non-planning impulsivity—were consistent with healthy population estimates and yielded no significant group differences in any subdimension.

#### Controlled Oral Word Association Test

The total number of words produced for letters F, A, and S were summed to create a composite verbal fluency score, while the Animal Naming was used to assess semantic memory. No significant between groups differences were observed for either verbal fluency or semantic fluency.

#### Iowa Gambling Task

**Figure 1:**
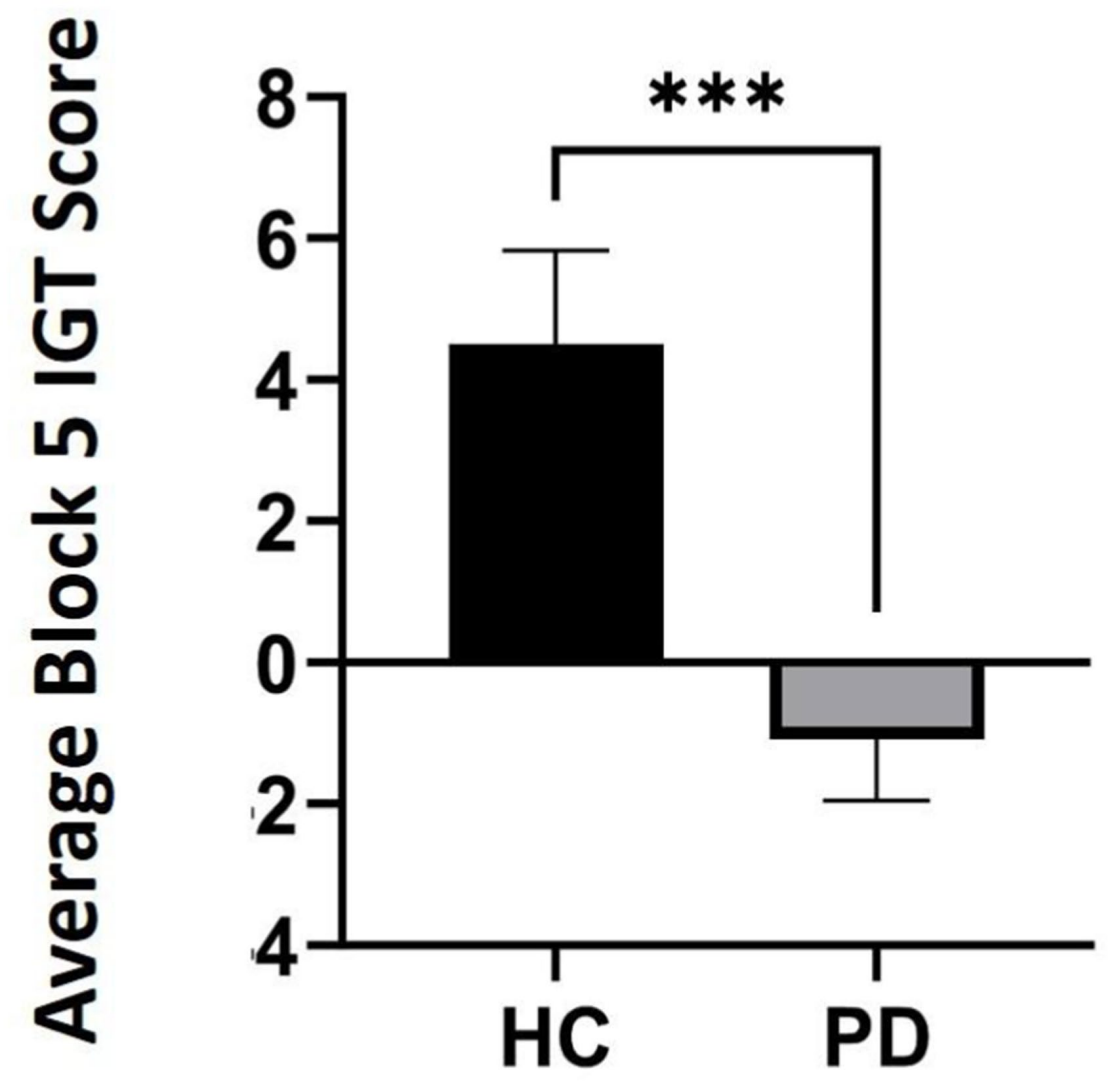
Average Iowa Gambling Task (IGT) scores in block 5 comparing participants with Parkinson’s disease (PD) to healthy controls (HC) by the end of the task (*p* = 0.008). *** refers to statistical significance at the *p* = 0.008 level.

**Figure 2:**
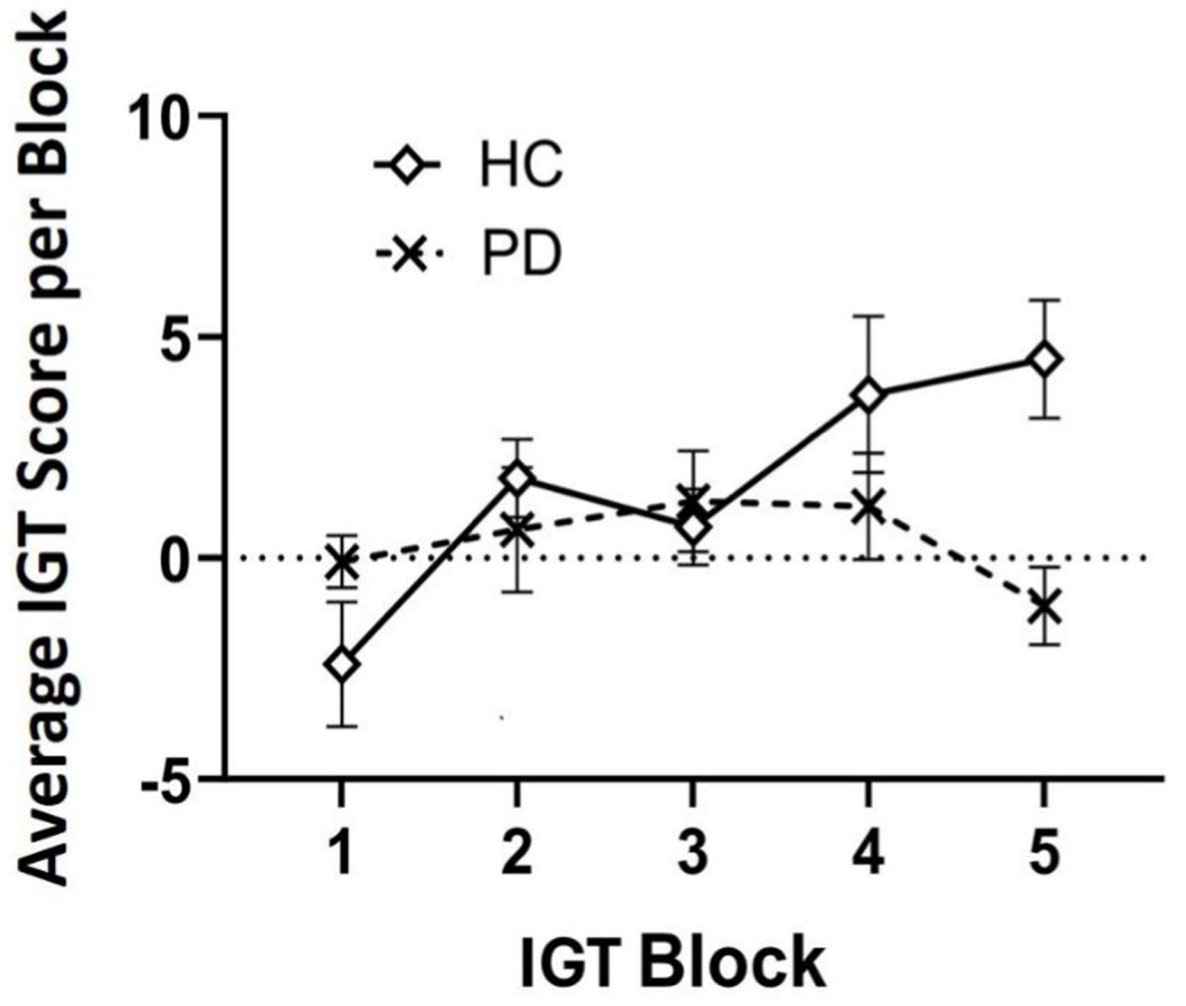
Average Iowa Gambling Task (IGT) score analyzed over time in five 20-trial blocks (100 trials total). By block 5, results suggest individuals with Parkinson’s disease (PD) maintained a disadvantageous selection strategy by the end of the task compared to healthy controls (HC).

Outlier analyses identified one extreme value for total winnings scores on the IGT in the PD group, thus analyses were conducted on *N* = 24 PD and *N* = 20 Controls. Results revealed that a group effect approached significance [*F* (43) = 3.11, *p* = 0.08], suggesting that the PD group accumulated fewer total winnings than controls. To investigate IGT performance on a more precise level, we conducted a general linear model (two-way ANOVA) across the task broken up into five 20-trial blocks to determine deck-specific responding per block that could reveal problematic learning behaviors over time. Results indicated a significant main effect of block [*F* (3, 39) = 3.08, *p* = 0.03, *η*_p_^2^ = 0.24]. Interestingly, this was qualified by a block by group interaction, [*F* (4, 39) = 2.77, *p* = 0.04, *η*_p_^2^ = 0.22]. Specifically, average block scores by the end of the task (block 5) were significantly worse than healthy controls, thus PD participants were persistently making disadvantageous as opposed to advantageous choices [one-way ANOVA: *F* (43) = 7.69, *p* = 0.008 *η*_p_^2^ = 0.15] (see Table 3 and Figures 1, 2).

#### Eye movements during the IGT

Eye movement results are listed in Table 4. Recall that we analyzed several eye tracking metrics: average fixation count, average fixation duration, average saccade count, and average blink count, during the entirety of the IGT. The mean trial completion times for individual trials were highly variable in the present study given the self-paced nature of the task (Range = 904.80–8818.30 ms, M = 1628.20 ms). We therefore analyzed our eye movement data based upon mean participant responses across trials.

**Table 4:** Eye movement results.

	PD group	HC group			
Eye movement	Mean (SD)	Mean (SD)	*F*	*p*	*η* ^2^
Fixation count	5.80 (2.5)	5.17 (2.3)	0.78	0.381	0.02
Fixation duration	336.37 (92.0)	308.94 (114.8)	0.79	0.390	0.02
Saccade Count	5.05 (2.6)	4.46 (2.1)	0.69	0.409	0.02
BLINK count	0.19 (0.3)	0.67 (0.4)	22.37	**<0.001**	0.37

Eye movements represent the mean number of observations averaged across 100 trials for the IGT. Fixation duration is reported in milliseconds. Bold values represent statistically significance group differences based upon the threshold of *p* <0.05.

#### Fixation count

Groups did not differ significantly, thus suggesting that PD participants and controls exhibited an equivalent number of fixations during the IGT.

#### Fixation duration

Similarly, average fixation duration did not differ significantly between groups during the IGT. Based upon our observation of a numerical trend, we conducted a series of exploratory partial correlations between fixation durations and mean performance during each of the five IGT blocks controlling for group. While fixation durations were not significantly correlated with performance in blocks 1 or 4 [rPLS (42) = −0.16, *p* = 0.30; rPLS (42) = 0.03, *p* = 0.81, respectively], they were significantly correlated with block 2 [rPLS (42) = 0.35, *p* = 0.02], block 3 [rPLS (42) = 0.34, *p* = 0.02], and block 5 [rPLS (42) = 0.37, *p* = 0.01]. These novel findings suggest a unique and revealing relationship between eye fixation durations and the ability (or inability) to learn and implement advantageous decision-making strategies.

#### Saccade count

Group differences in saccades were not significant, suggesting that PD participants and controls exhibited an equivalent number of saccadic movements during the IGT.

#### Blink count

A one-way ANOVA on average blink count revealed a highly significant difference between groups. Individuals with PD exhibited a 72% reduction in eye blinks during the IGT compared to the healthy control group, resulting in a very large effect size. These blink count data (in Table 4) are an extremely valuable outcome that underscores the role of striatal dopamine function in PD, given that this anatomical area and neurotransmitter are involved in reinforcement-based learning over time.

## Discussion

We compared early-stage to mid-stage individuals with PD to age-matched healthy controls using a novel combination of the IGT coupled with eye tracking and additional cognitive and motor tests. As expected, PD participants experienced higher adverse impacts on daily living and more motor complications than matched healthy controls. Individuals with PD also exhibited significantly longer completion times on both Trail Making Tests, significantly worse decision-making over time in the IGT, and a significant reduction in blink rate despite similar eye fixation and saccade rates.

In accordance with our first prediction, participants with PD scored higher on each of the three UPDRS subscales than controls, and completion times on our measure of attention and cognitive flexibility were significantly longer in the PD group compared to controls. Additionally, participants in the PD group exhibited a higher number of errors with increased task demands compared to their control counterparts. This pattern is consistent with established norms (Tombaugh, 2004) and supports previous findings of impaired executive function in PD (Aron et al., 2004; Dulas and Duarte, 2014; Dulas and Duarte, 2016; Kudlicka et al., 2011). Our measures of impulsivity and verbal fluency revealed similar scores between groups, therefore it is possible that our PD sample had not yet reached diagnostic thresholds for impulse control disorders (Giovannelli et al., 2023) or impoverished oral semantic fluency (Hedman et al., 2022).

With respect to our prediction about IGT performance, we did observe an overall difference in net scores between groups that approached significance. This is in accordance with pattern in the previous literature of diminished decision-making in PD patients compared to healthy controls (Castrioto et al., 2015; Xi et al., 2015; Kobayakawa et al., 2010; Kobayakawa et al., 2017). Upon closer examination of decision-making across task blocks (Kobayakawa et al., 2017), we discovered a unique pattern. By the fifth and final block of trials, individuals with PD had exhibited a net negative score (i.e., losses), whereas the healthy controls had accumulated a positive score (winnings), and this difference was highly significant. The current literature on IGT in PD presents mixed findings regarding behavioral performance (Salvatore et al., 2021). Some studies report impaired performance in PD compared to healthy controls, whereas others do not (e.g., Poletti et al., 2012). Our findings underscore the importance of evaluating decision-making changes over time within a task, rather than relying solely on aggregate performance. The fact that PD participants maintained a disadvantageous deck selection strategy by the task’s end, potentially reflects insensitivity to aversive outcomes in reward-based learning (Salvatore et al., 2021; Kobayakawa et al., 2010; Kobayakawa et al., 2017). This aligns with the hypothesis that dopaminergic and noradrenergic changes in PD, particularly involving the substantia nigra, basal ganglia, and prefrontal cortex functionally manifest as an insensitivity to negative feedback, thus impairing error correction.

With respect to our eye movement predictions, we observed a highly significant 70% reduction in eye blinks during decision-making in PD participants. This is in accordance with prior research documenting reduced task-independent spontaneous blink rates in mid-stage PD patients (Iwaki et al., 2019; Kimura et al., 2017). A substantial reduction in blink rate may reflect disrupted dopaminergic and norepinephrinergic function, which plays a role in both motor control and cognitive processing (Van Slooten et al., 2019; Jongkees and Colzato, 2016; Willett et al., 2023; Redgrave et al., 2010). Additionally, decreased dopaminergic activity could contribute to impaired motor execution in regions innervated by the corticobulbar tract. This pathway involves regulating movements of the face, head, and neck by influencing the cranial nerves, including those that control the extraocular muscles (cranial nerves III, IV, and VI). While this pathway does not directly regulate eye blinks, its interaction with brainstem regions could indirectly impact voluntary eye movements. Notably, a lower blink rate may contribute to the characteristic hypomimia, or “facial masking,” commonly observed in PD, which has psychosocial implications for both patients and caregivers (Bianchini et al., 2024). Importantly, these findings support the utility of blink rate as a diagnostic marker, particularly during cognitive tasks such as the IGT in the early stages of PD (Van Slooten et al., 2019; Tao et al., 2020). Researchers should reconsider the exclusion of eye blinks as artifacts during eye-tracking analyses given their strong association with dopaminergic function. Contrary to our expectations, we did not find significant group differences in the average number of saccades, fixations, fixation duration, or pupil dilation. However, exploratory analyses revealed significant correlations between fixation duration and decision-making performance in the middle and final blocks, suggesting a potential cognitive interaction that warrants further investigation.

This study is the first, to our knowledge, to integrate eye tracking with the IGT in individuals with PD. Our findings reveal nuanced cognitive and physiological distinctions that may serve as early indicators of disease progression. Subtle strategy shifts in decision-making over time, coupled with distinct eye movement patterns, suggest that a combined approach may enhance diagnostic sensitivity in early PD. Future research should prioritize multimodal assessments that integrate cognitive testing (including decision-making tasks as well as working memory and executive functioning tasks) with non-invasive physiological measures like eye tracking. Such an approach could provide a more comprehensive characterization of cognitive dysfunction in PD and facilitate earlier detection of subtle impairments. This early detection may have valuable practical implications. For instance, a recent review by Salvatore et al. (2021) highlights that the neurobiological changes in dopaminergic and noradrenergic innervation at the earliest stages of PD lend themselves for interrogation by the IGT in detecting early PD pathology and predicting treatment adherence. Notably, non-strategic decision-making in PD may correlate with non-compliance or self-discontinuation of therapeutic regimens. Given that up to 70% of PD patients opt to discontinue treatment despite life-threatening risks (Straka et al., 2019; Daley et al., 2012), these findings highlight a critical deficit in evaluating long-term consequences in decision-making. Our results also suggest that reduced eye blinks may represent a prodromal symptom that can be objectively measured before the onset of profound motor symptoms. Furthermore, it is possible that a reduction in eye blinks, facial masking, and apparent apathy are interconnected. Understanding the relationship between these symptoms is crucial for understanding the progression of PD.

Our study is not without limitations. Participants were a well-educated, high-functioning, and racially/ethnically homogeneous subset of the population with access to medical care. Future research should aim for greater demographic diversity in sample recruitment. Additionally, our relatively small sample size may have limited statistical power to detect group differences in saccadic behavior and fixation patterns. Our brief measure of impulsivity may also have lacked sensitivity; therefore, future studies should incorporate PD-specific assessments of impulse control disorders (Giovannelli et al., 2023). Future work should strive to balance sex of patient and control samples. Previous literature suggests that healthy men tend to perform better than healthy women on the task (Zanini et al., 2024), however we are not aware of any normative sex data on IGT performance of individuals with PD.

Finally, we suggest that creating a standardized version of the IGT including a pre-stimulus baseline adapted for eye tracking would enhance the precision of oculomotor analyses. The current study was limited by the short duration of each trial and the variability of self-paced responses, which restricted fine-grained analyses of discrete eye movement phases (e.g., initial presentation, deck selection, feedback).

In conclusion, the IGT holds promise for assessing cognitive changes in Parkinson’s disease, particularly for evaluating decision-making abilities. As the disease progresses, subtle changes in cognition, such as executive function, decision-making, and risk assessment, may decline. This has key implications for daily life and treatment decisions. Considering the currently projected doubling of individuals who will develop the disease in their lifetime (Obeso et al., 2017), there is valuable utility in combining cognitive assessments with physiological markers to gain a more comprehensive understanding. Integrating sensitive measures such as eye tracking with a range of cognitive tests, including decision-making, working memory, and other executive function tasks, alongside other disease markers may provide a simple yet powerful method for detecting dysfunction early in disease progression. This multimodal approach could improve characterization of disease progression and enable earlier intervention before significant motor symptoms emerge.

## Data Availability

The raw data supporting the conclusions of this article will be made available by the authors, without undue reservation.
